# Understanding Sociodemographic Factors among Hispanics Through a Population-Based Study on Testicular Cancer in Mexico

**DOI:** 10.1007/s40615-023-01859-0

**Published:** 2023-11-14

**Authors:** Juan Alberto Ríos-Rodríguez, Michel Montalvo-Casimiro, Diego Ivar Álvarez-López, Nancy Reynoso-Noverón, Berenice Cuevas-Estrada, Julia Mendoza-Pérez, Miguel A. Jiménez-Ríos, Talia Wegman-Ostrosky, Pamela Salcedo-Tello, Anna Scavuzzo, Clementina Castro-Hernández, Luis A. Herrera, Rodrigo González-Barrios

**Affiliations:** 1 Unidad de Investigación Biomédica en Cáncer, Instituto Nacional de Cancerología-Instituto de Investigaciones Biomédicas, UNAM, Mexico City, 14080 México; 2 Tecnologico de Monterrey, Escuela de Medicina y Ciencias de La Salud, Monterrey, 64710 México; 3 Unidad de Epidemiología en Cáncer, Instituto Nacional de Cancerología, Mexico City, 14080 México; 4 Department of Translational Molecular Pathology, The University of Texas MD Anderson Cancer Center, Houston, TX 77030 USA; 5 Departamento de Urología, Instituto Nacional de Cancerología, Mexico City, 14080 México; 6 Departamento de Bioquímica, Facultad de Medicina, UNAM, Mexico City, 04510 México; 7 Departamento de Biología Celular, Facultad de Ciencias, UNAM, Mexico City, 04510 México

**Keywords:** Testicular cancer, TGCT, Hispanic population, Health disparities, Incidence trends, Cancer survival

## Abstract

Testicular cancer (TCa) is a rare malignancy affecting young men worldwide. Sociodemographic factors, especially socioeconomic level (SEL) and healthcare access, seem to impact TCa incidence and outcomes, particularly among Hispanic populations. However, limited research has explored these variables in Hispanic groups. This study aimed to investigate sociodemographic and clinical factors in Mexico and their role in health disparities among Hispanic TCa patients. We retrospectively analyzed 244 Mexican TCa cases between 2007 and 2020 of a representative cohort with diverse social backgrounds from a national reference cancer center. Logistic regression identified risk factors for fatality: non-seminoma histology, advanced stage, and lower education levels. Age showed a significant trend as a risk factor. Patient delay and healthcare distance lacked significant associations. Inadequate treatment response and chemotherapy resistance were more likely in advanced stages, while higher education positively impacted treatment response. Cox regression highlighted non-seminoma histology, below-median SEL, higher education, and advanced-stage survival rates. Survival disparities emerged based on tumor histology and patient SEL. This research underscores the importance of comprehensive approaches that integrate sociodemographic, biological, and environmental factors to address health disparities improving outcomes through personalized interventions in Hispanic individuals with TCa.

**Supplementary Information:**

The online version contains supplementary material available at 10.1007/s40615-023-01859-0.

## Introduction

Testicular cancer (TCa) is a rare malignancy, comprising a mere 1 to 2% of all male cancers, with a steady increase in incidence over the past few decades [1]. While racial differences in TCa rates have been observed, there is a concerning upward trend across all ethnicities, necessitating further investigation and comprehension of this disease [2, 3]. The rising incidence is particularly significant as TCa primarily affects young men aged 15 and 44, rendering it the most prevalent neoplasia in this age group [4].

A study conducted by Ghazarian from 2001 to 2016 sheds light on the incidence of testicular germ cell tumors (TGCTs) among diverse racial and ethnic groups. Non-Hispanic whites (NHW) exhibited the highest incidence rate, followed by Hispanics, American Indian/Alaska Natives (AI/AN), Asian/Pacific Islanders (A/PI), and non-Hispanic Blacks (NHB). Importantly, the incidence of TGCTs increased significantly across all racial and ethnic groups during this period, with the most notable increase observed among A/PIs and Hispanics [5]. Projections suggest that by the year 2026, Hispanics will have the highest TGCT rate across all racial/ethnic groups in the USA, highlighting the pressing need for increased attention to this population. Moreover, this trend may extend to countries with predominantly Hispanic populations, underscoring the importance of addressing this health concern on a broader scale [6]. In the sociodemographic context, Hispanics constitute a diverse pan-ethnic group with genetic origins primarily stemming from Western Europe, West Africa, and East Asia. Their ancestral backgrounds exhibit a wide range of structures, ethnicities, and nationalities across Ibero-American regions, resulting in intricate population subgroups. In this study, we specifically explore a subset within the broader Hispanic community, namely the Mexican-Hispanic subpopulation, which shares common ancestral cultural influences [7].

Recent research suggests that sociodemographic factors, such as socioeconomic level (SEL) and healthcare accessibility, exert an influential role in the observed increases in TCa incidence and mortality rates among Hispanics. Studies have shown that Hispanic men residing in low SEL, or high enclave neighborhoods face a higher risk of late-stage diagnosis for both seminomas and non-seminomas. Additionally, Hispanic adolescents and young adults (AYAs) appear to have poorer overall survival rates following non-seminoma testicular cancer [8, 9]. Geographically, variations in TCa burden and mortality rates have been observed, with higher rates reported in countries with a low human development index (HDI), suggesting the impact of socioeconomic factors on healthcare accessibility [1, 10, 11].

This phenomenon could have international implications. A study conducted in New Mexico revealed increased incidence rates among the Hispanic population residing in the border region between the USA and Mexico. Surprisingly, these elevated rates remain unexplained within the current understanding of established risk factors. Notably, this population exhibited poorer outcomes, characterized by lower 5-year survival rates and a higher proportion of regional and distant cancer cases at the time of diagnosis [12].

In the specific context of Mexico, TCa has emerged as a significant public health concern. In 2020, Mexico ranked fifth in the Latin American Caribbean region in terms of the highest incidence of TCa and first in terms of associated mortality when adjusting for ages 15 to 39. However, these statistics may potentially underestimate the actual incidence and mortality rates [5, 13] implying that the true numbers could be even higher. Projections generated by the Cancer Tomorrow tool suggest that by the year 2040, Mexico is expected to rise in global rankings. Within the context of AYAs population, maintaining the highest mortality rates within the Caribbean and Latin America region [1, 14].

Despite limited research on representative samples in Mexico, the available studies align with international findings regarding TCa among Hispanics. Factors such as the response to chemotherapy, manifestation of progressive disease symptoms, hospital type, and education level have been identified as potential contributors to disease outcomes. However, further research is needed to comprehend the specific epidemiology and risk factors within the Mexican population [15–17].

Previously, at the Instituto Nacional de Cancerología (INCan), the national reference cancer center in Mexico, testicular cancer has been described as the most common neoplasm in the genitourinary system from 1985 to 1994 [18], and the second most prevalent in men in the years 2007 to 2009 in central Mexico, only after prostate cancer [19]. According to the hospital records, the INCan treated around 2600 TCa patients between the years 2007 and 2020, which exhibited a statistically significant distribution similar to the representative cohort of 244 patients provided in this article.

Taking these insights into account, this article explores the influence of sociodemographic variables (age, SEL, educational level, patient delay, and distance to tertiary referral hospital) and clinical features (histology, stage, and initial symptom), aiming to establish associations between RECIST-based treatment response, chemoresponse, and survival in TGCT patients treated at the INCan. This article offers a novel perspective to the study of health disparities within Mexican-Hispanic patients in a representative population approach.

## Methods

A retrospective analysis was conducted on a representative cohort of 244 patients diagnosed with TCGT at the INCan between 2007 and 2020. The age range of these patients was between 17 and 60 years. The included criteria comprise patients who received equitable access to healthcare services, regardless of sociodemographic variables and the patient’s insurance status, and were treated in accordance with stratification and standardized management modalities according to NCCN Clinical Practice Guidelines in Oncology. Underwent radical orchiectomy and were histopathologically classified based on the WHO Classification of Tumors of the Urinary System and Male Genital Organs. The staging was performed according to the American Joint Committee on Cancer (AJCC) guidelines, and classification was based on the prognostic groups of the International Collaborative Group of Germ-Cell Tumors (IGCCCG). The chemotherapy regimen as first-line included bleomycin, etoposide, and cisplatin (BEP), three to four cycles, to which subsequent lines and adjuvant treatments may be added as needed. Individuals with incomplete clinical records or those who did not complete their treatment at INCan were excluded.

The research for this study was derived from a previously approved protocol by the research committee protocols (012/031/ICI) and the ethics research committee of the INCan, with protocol numbers CEI/783/12 and CEI/052/22. The study strictly adhered to the principles outlined in the Helsinki Declaration and was conducted following institutional guidelines, including obtaining informed consent from the participants.

Age, education level, initial symptom, SEL, patient delay (the time between the first manifestation and the diagnostic), distance to tertiary referral hospital (DTRH), and certain characteristics of the medical condition (histology, stage, and treatment modality) were all recollected from medical records and examined in relation to vital status (case fatality risk). We also analyzed the relationship of these variables with the patient’s response to treatment and chemo-response.

After treatment, objective response rates were evaluated using CT scans within 6 weeks of completing the BEP chemotherapy, based on RECIST criteria version 1.1. Patients classified as having a complete treatment response (CTR) indicated that all lesions and pathologic lymph nodes had disappeared prior to treatment, while inadequate treatment response (ITR) described patients who had a partial response, stable disease, or progressed despite treatment. The chemoresponse group was defined as Resistant and Sensitive. Platinum resistance was defined as a disease that consistently progressed under platinum-based chemotherapy, progressive disease (relapse or incomplete response) after one or more complete platinum-based regimens within the first 2 years of follow-up, viable (non-teratomatous) disease in a post-chemotherapy surgical sample, or persistent or increasing tumor markers 4 weeks after a complete four-cycle chemotherapy regimen.

SEL was determined using a National Inter-Institutes of Health parameter, which had been extracted from the clinical record on the social worker section, which in turn was obtained from a direct interview with the patient. Primarily, the SEL rubric encompassed family type, income for housing, patient residency, employment, and food income, where patients were categorized into 7 levels depending on their score, from 0 (0–15) to 6 (85–100). Additionally, an experienced social worker used the SEL rubric to assign an alternative level known as the Perceived Socioeconomic Level (PSEL), based on the patient’s context and characteristics at the time of the interview. For analysis purposes, we calculated the median, and dichotomized these variables into below (0–2) and above the median (3–6) as described in the Supplementary Table 1.

The variables of age, DTRH, diagnostic delay, treatment modality, stage, initial symptom, education levels, socioeconomic level, perceived socioeconomic level, patient’s residence, and risk classification underwent a descriptive analysis and were subsequently grouped and separated by the histology in a univariate analysis, using logistic regression and chi-square test.

In order to evaluate the most significant variables to lethality, we run a univariate analysis. For non-parametric continuous variables (age, DTRH and patient delay), we performed logistic regressions. The Fisher exact test was used for Histology, Stage, and Initial symptom variables, while Chi-Square tests were used for the remaining variables, additionally, odds ratios were calculated through cross-tables and logistic regression (Supplementary Table 2). Moreover, we evaluated the association between educational level and delay to diagnosis using a Kruskal–Wallis test (Supplementary Table 3).

Subsequently, we conducted logistic regression analyses to study the relationship between the previous significant variables and the clinically relevant ones (SEL, patient delay, DTRH, age, education level, initial symptom, histology, and stage) while comparing vital status. Following that, in other multivariate analyses, we replaced the SEL variable with perceived socioeconomic level (PSEL) and, subsequently substituted by the place of residency, to avoid confounding variables. We also analyzed the most relevant factors for treatment response and chemo-response as dependent variables. Goodness of fit analysis through Hosmer–Lemeshow test was performed.

We conducted a Cox regression analysis and employed Kaplan–Meier curve to assess their impact on survival time, from the beginning of their diagnosis until completing 5 years of follow-up. Patients who did not meet the criteria were censored.

## Results

**Fig. 1:**
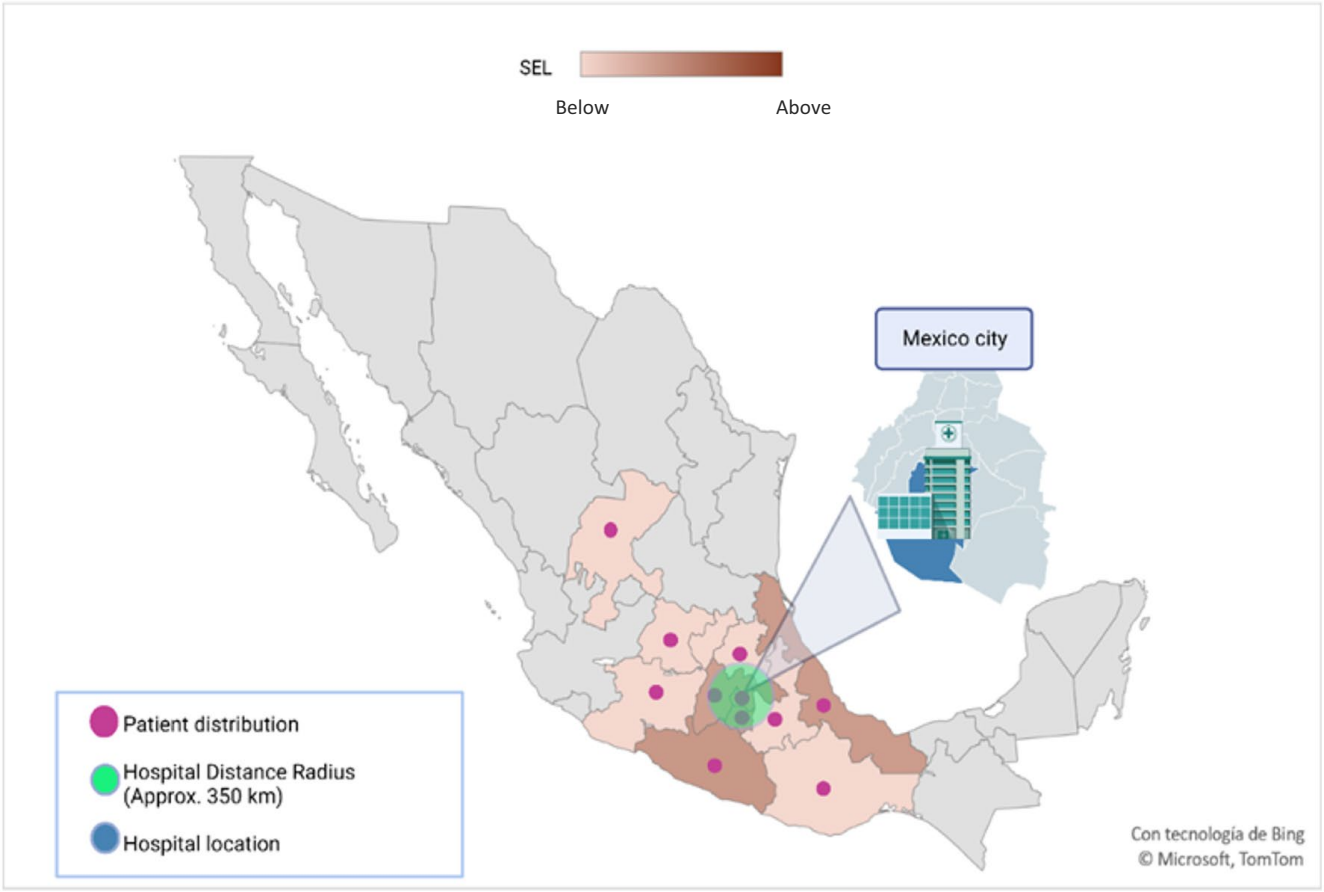
Distribution of TGCT patients based on SEL

We recruited a cohort of 244 Mexican patients at the INCan, with a median follow-up duration of 2 years (IQR: 1–10 years), during the period from 2007 to 2020, of which 195 cases (79.9%) were non-fatal whereas 47 cases (20.1%) were fatal. The patients are geographically dispersed within the central and southern regions of the country, demonstrating a diverse socioeconomic profile. Their distances from healthcare facilities vary significantly, ranging from 2 to 641 km away from the medical facilities (Fig. 1). Among them, 56 patients (23%) underwent a local approach (orchiectomy) while 188 patients (77%) received adjuvant chemotherapy. Of the patients, 128 (68,1%) were described as chemo-sensitive, while 60 patients (31.9%) were cataloged as chemo-resistant. Notably, around 50% of non-seminoma patients enter this chemo-response category. In addition, the prevalence of non-seminoma histology (N = 151, 61.9%) is higher compared to seminoma (N = 93, 38.1%). Other relevant clinical variables were compared and summarized in Table 1.

**Table 1:** Descriptive analysis of TGCT patients compared by histology

	Total 244 (100%)	Seminoma 93 (38.1%)	Non-seminoma 151 (61.9%)	*p*-value^a^
Age, years median (IQR)	26 (22–32)	30 (22–36)	24 (19–27)	< 0.001^b^
DTRH, kilometers median (IQR)	36 (15.8–76.6)	36 (10.5–100.5)	36 (11.5–100.5)	0.587^b^
Diagnostic delay, months median (IQR)	3 (1.6–7)	4 (1–7)	3 (0.5–5.5)	0.079^b^
Treatment modality Orchiectomy Adjuvant chemotherapy	56 (23) 188 (77)	11 (11.8) 82 (88.2)	45 (29.8) 106 (70.2)	0.001
Chemoresponse Resistant Sensitive	60 (31.9) 128 (68.1)	9 (11) 73 (89)	51 (48.1) 55 (51.9)	< 0.001
Treatment response Inadequate Complete	69 (28.3) 175 (71.7)	12 (12.9) 81 (87.1)	57 (37.7) 94 (62.3)	< 0.001
Histological subgroup Teratoma Embryonal Carcinoma Choriocarcinoma Mixed Pure seminoma	8 (3.3) 8 (3.3) 2 (0.8) 133 (54.1) 93 (38.1)	- - - - 93 (100)	8 (5.3) 8 (5.3) 2 (1.3) 133 (88.1) -	-
Stage Locoregional (I, II) I II Advanced (III)	153 (62.7) 46 (18.9) 107 (43.9) 91 (37.3)	80 (86) 22 (23.7) 58 (62.4) 13 (14)	73 (48.3) 24 (15.9) 49 (32.5) 78 (51.7)	< 0.001
Initial symptom Testicular pain Testicle swelling Mixed symptomatology Symptoms associated with metastasis	16 (6.6) 132 (54.1) 57 (23.4) 39 (16)	9 (9.7) 51 (54.8) 24 (25.8) 9 (9.7)	7 (4.6) 81 (53.6) 33 (21.9) 30 (19.9)	0.095
Education levels Elementary Middle school High school Higher grade	36 (14.8) 70 (28.7) 77 (31.6) 61 (25)	10 (10.8) 26 (28) 24 (25.8) 33 (35.5)	26 (17.2) 44 (29.1) 53 (35.1) 28 (18.5)	< 0.05
Socioeconomic level Median (IQR) Below median Above median	2 (1–3) 164 (67.2) 80 (32.8)	59 (63.4) 34 (36.6)	(69.5) 46 (30.5)	0.325
Perceived socioeconomical level Below median Above median	166 (68) 78 (32)	58 (62.4) 35 (37.6)	108 (71.5) 43 (28.5)	0.136
Patient’s residence Local Out-of-town	104 (42.6) 140 (57.4)	40 (43) 53 (57)	64 (42.4) 87 (57.6)	0.923
Risk classification Good Intermediate Poor	158 (64.8) 33 (13.5) 53 (21.7)	82 (88.2) 11 (11.8) -	76 (50.3) 22(14.6) 53 (35.1)	< 0.001

*IQR*, Interquartile range

*Ref*, Referential variable

^a^ Calculated using Chi-Square

^b^ Calculated using Kruskal–Wallis test

* Significant at *p* < 0.05

** Significant at *p* < 0.001

Our findings revealed significant associations between several factors, including SEL below median, non-seminoma histology, symptoms related to metastasis, educational levels, and advanced stage. However, we did not observe significant statistical associations with patient delay, distance, and age (Table 2).

**Table 2:** Analysis of variables for fatal and non-fatal cases: Univariate, Logistic regression, and Cox regression

	Fatal cases	Non-fatal cases	Univariate analysis		Adjusted analysis (Logistic regression)		Adjusted analysis (Cox regression)	
	*n* = 49 (20.1%)	*n* = 195 (79.9%)	OR (95%CI)	*P*-value^a^	OR (95%CI)	*P*-value^a^	HR (95%CI)	*P*-value^d^
Age in years, median (IQR)	25 (21–29)	26 (22–32)	0.97 (0.93–1.01)	0.145^a^	1.07 (0.98–1.15)	0.092	1.05 (1–1.11)	0.051
Delay in months, median (IQR)	3 (2–7)	3 (1.4–7)	1.002 (0.97–1.03)	0.897^a^	0.98 (0.94–1.01)	0.131	0.98 (0.96–1.01)	0.13
Histology Seminoma Non-seminoma	3 (6.12) 46 (93.88)	90 (46.15) 105 (53.85)	Ref 13.14 (3.98–67.75)	< 0.001^c^	Ref 9.56 (1.97–46.37)	< 0.05	Ref 6.02 (1.61–22.47)	< 0.05
Stage Locoregional Advanced	3 (6.12) 46 (93.88)	150 (76.92) 45 (23.08)	Ref 51.11 (15.04–263.91)	< 0.001^c^	Ref 29.09 (7.69–109.92)	< 0.001	Ref 31.22 (7.22–134.98)	< 0.001
Initial symptom Testicular pain Testicle swelling Mixed symptoms Metastasis related	1 (2.04) 19 (38.78) 8 (16.33) 21 (42.86)	15 (7.69) 113 (57.95) 49 (25.13) 18 (9.23)	Ref 2.52 (0.29–19.27) 2.44 (0.28–21.73) 17.5 (1.49–166.9)	0.70^c^ 0.67^c^ < 0.001^c^	Ref 2.23 (0.17–29.52) 2.07 (0.14–29.55) 4.66 (0.33–64.93)	0.54 0.59 0.25	Ref 1.73 (0.21–14.02) 2.34 (0.27–20.11) 2.33 (0.29–18.86)	0.61 0.44 0.42
Socio-economic level Median (IQR) Above median Below median	2 (2–3) 9 (18.37) 40 (81.63)	71 (36.41) 124 (63.59)	Ref 2.54 (1.13–6.30)	< 0.05^b^	Ref 2.45 (0.774–7.75)	0.128	Ref 2.62 (1.12–6.11)	< 0.05
Education level Elemental Middle school High School Higher grade	18 (36.73) 8 (16.33) 19 (38.78) 4 (8.16)	18 (9.23) 62 (31.79) 58 (29.74) 57 (29.23)	Ref 0.13 (0.04–0.38) 0.33 (0.14–0.78) 0.07 (0.02–0.28)	< 0.001 < 0.01 < 0.001^c^	Ref 0.25 (0.071–0.85) 1.120 (0.320–3.91) 0.24 (0.050–1.12)	< 0.05 0.860 0.070	Ref 0.419 (0.17–1.01) 1.161 (0.53–2.55) 0.259 (0.073–0.92)	0.053 0.710 < 0.05

*IQR*, interquartile range

*Ref*, referential variable

^a^ Calculated using a logistic regression

^b^ Calculated using a chi-square test of independence

^c^ Calculated using a Fisher’s exact test

^D^ Calculated using a Cox regression

* Significant at *p* < 0.05 ** Significant at *p* < 0.001

In the multivariate-adjusted analysis (logistic regression), certain factors remained statistically significant while others did not. Specifically, non-seminoma histology (OR: 9.565, *p*-value < 0.05), advanced stage (OR: 29.085, *p*-value < 0.001), and middle school (OR: 0.246, *p*-value < 0.05) retained their statistical significance (for reference categories of each measure association see Table 2). However, SEL, high school and higher education levels, and initial symptoms lost their significance in contrast to univariate analysis. Patient delay, DHTR, and age did not show any significant associations (Table 2). Importantly, as we previously described, these results remained consistent even when SEL was replaced with PSEL and patient residency. Moreover, the trend was consistent when the DTRH variable was introduced in the model (data not shown).

To assess the impact of the most relevant clinical and statistical variables over therapeutic response and differentiate the variables associated with disease progression, we also conducted a model using the treatment response group and chemo-response as dependent variables. This analysis revealed a significant relationship between the advanced stage (OR = 29.02, *p*-value < 0.001) and education level (higher education OR = 0.120, *p*-value < 0.05) with an inadequate response. However, SEL, histology, distance, age, and patient delay did not exhibit any significant correlations with the treatment response group. Regarding chemo-response, it showed a significant relationship only with the advanced stage (OR: 20.29, *p*-value < 0.001) as the dependent variable (Supplementary Table 2). In the histology model, the elemental education level was found to be related to non-seminomas (OR: 4.738, *p*-value < 0.005) and age (OR: 0.879, *p*-value < 0.001) (Supplementary Table 3). Patient delay did not have a statistical difference between education levels (Supplementary Table 4).

The Cox regression model revealed the impact of variables on survival time, non-seminoma histology (HR: 6.019, *p*-value < 0.05), below median SEL (HR: 2.618, *p*-value < 0.05), higher education (HR: 0.259, *p*-value: < 0.05), and advanced stage (HR: 31.22, *p*-value: < 0.05) were all statistically significant factors affecting survival time. Additionally, age (HR: 1.054, *p*-value: 0.051) and middle school (HR: 0.419, *p*-value: 0.053) showed tendencies toward statistical significance (Table 2).

**Fig. 2:**
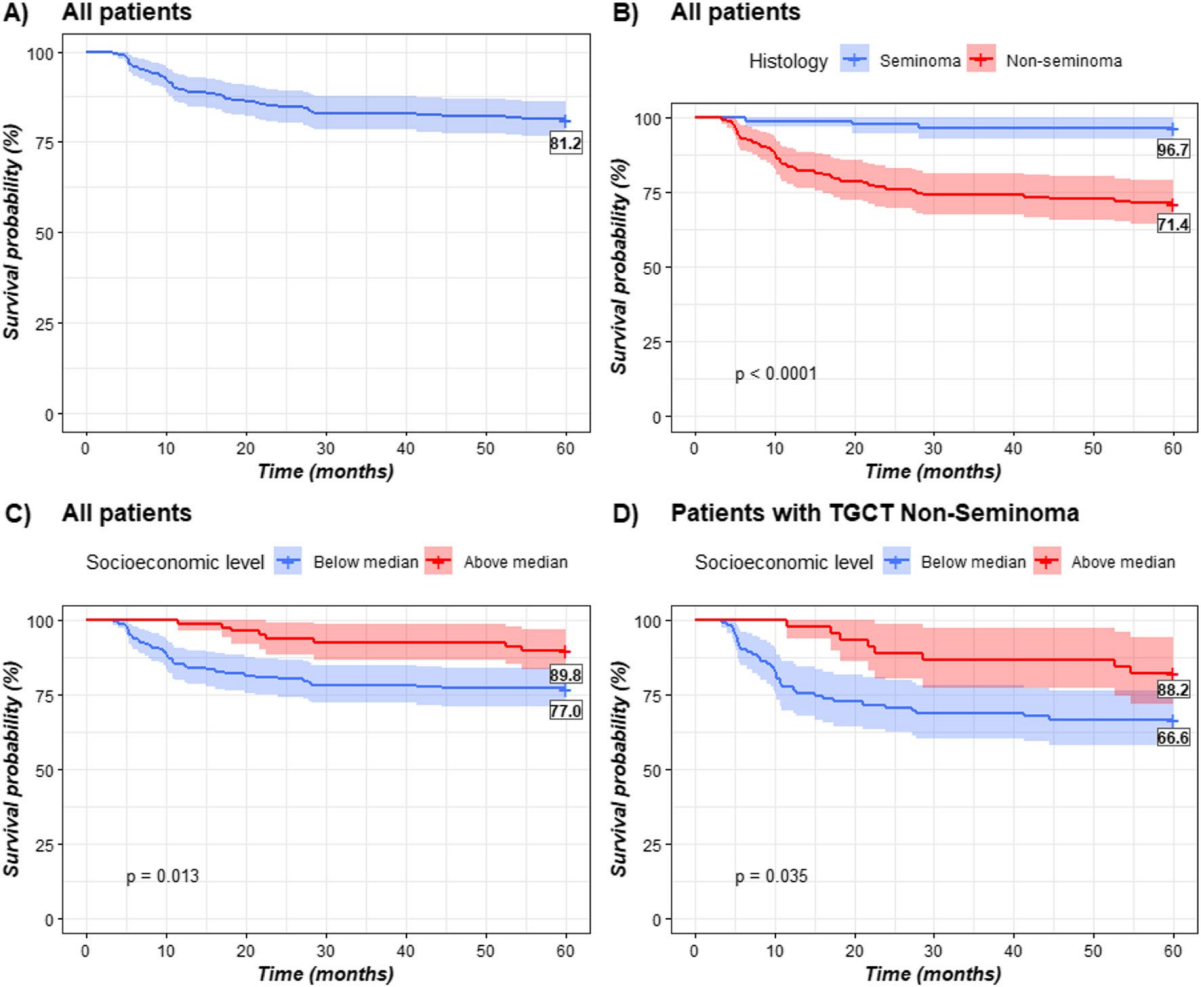
Estimation of survival rates in TGCT patients with Kaplan–Meier curves adjusted by SEL and Histology group

The Kaplan–Meier curves suggests an overall survival (OS) rate of 81.2%, I.C. 95% (Fig. 2A), with variations observed based on histology (seminoma: 96.7%, non-seminoma: 71.4%, LR: 23.4, *p*-value: < 0.001), and SEL (above median: 89.8%, below median: 77%, LR: 6.22, *p*-value: 0.013) (Fig. 2B and C). The OS rate among non-seminoma patients differs significantly with respect to SEL (Above median: 88.2%, below median: 66.6%, LR: 5.69, *p*-value: 0.035) as shown in Fig. 2D.

## Discussion

The present study was conducted at a single site, INCan between 2007 and 2020, and encompassed patients from different regions of the country with diverse socioeconomic positions (Fig. 1). Despite the lack of current and comprehensive epidemiological data on TCa in Mexico, the patients involved in this study offer valuable insights into the situation, making it the first of its kind with such characteristics. This study also employed a standardized parameter from the National Inter-Institutes of Health to measure SEL, a parameter previously validated in a representative healthcare facility in Mexico [13, 20, 21].

Historically, low SEL has been linked to a heightened risk for lower overall survival (OS) rates compared to international norms. Extensive literature highlights the link between Hispanics’ low rates of survival and their socioeconomic status [6, 12, 13, 20]. However, in the present study, we did not find a significant association of SEL, as a higher possibility variable for fatal cases. This suggests that the impact of SEL may be better understood in the context of its association with the duration of survival rather than as a factor intrinsically linked to the case-fatality risk of TCa. Consequently, this prompts us to suggest the possibility that there exist other variables apart from SEL which could contribute to elucidating the survival patterns observed in the disease within Hispanic populations. Importantly, socioeconomic level is a determinant of survival time rather than the case-fatality rate of the disease. Interestingly, in our study. As described in our results, patients with above median SEL surprisingly show a shorter survival time than expected [22].

Previous studies have demonstrated a correlation between low SEL and an increased likelihood of being diagnosed at advanced stages, which could be occurring in the phenomenon we observed in our study [9]. Nevertheless, even after accounting surveillance for sociodemographic variables like neighborhood and SEL in regression analyses, Hispanic men still exhibited diminished survival rates regardless of stage at diagnosis [23]. These variables appear to exert a similar influence across all ethnicities [24], particularly among AYAs residing in neighborhoods with middling to below average SEL. This underscores inherent distinctions in tumor biology that transcend SEL or PSEL considerations as the main driver of case-fatality risk [25–29].

Subsequently, an analysis was conducted to evaluate a possible association between the patient’s age at the time of diagnosis and its potential influence on the survival rate. Similar to the previous phenomenon, this was also not significant in the logistic regression model. However, the Cox regression reported a 5% increase in hazard risk per year for a low survival rate in TGCT. Previous studies have reported higher survival rates among AYAs compared to older men [30]. Derouen et al. demonstrated that AYAs aged 15–24 exhibit higher overall survival (OS) rates compared to individuals aged 25–39 with non-seminoma. This difference in age could be attributed to various factors, such as the lower prevalence of comorbidities among younger AYAs, variations in treatment approaches between pediatric and adult settings, or the greater resilience of younger patients in tolerating more rigorous treatment regimens [31]. Additionally, younger ages at diagnosis have been described as having a relatively higher risk of familial TCa compared to older individuals [30, 32, 33].

In previous works, Hispanics have been linked to a younger age at diagnosis. However, this does not protect them from being diagnosed at advanced stages, revealing a possible relationship between age and prognosis in different ethnic groups [34, 35]. Higher age at diagnosis seems to be associated with poorer outcomes due to acquired characteristics aging-related in the patient. Nonetheless, the presence of advanced stages among younger patients might imply that the tumor’s biological attributes, including histology and the stage during diagnosis, hold a more substantial influence over clinical outcomes [36]. Within our study, these variables, alongside educational attainment, demonstrated statistical significance in both models concerning the fatal outcome. This suggests that educational background acts as a safeguard against delayed diagnoses, exerting an even more pronounced impact than the patient’s age at the time of diagnosis.

In addition, Wynd et al. established that Hispanics with an education level below college were more hesitant to engage in testicular self-examination (TSE), which could potentially elucidate our findings regarding lethality and treatment response [37]. However, the relationship between stage and histology, in conjunction with education level, appears to be the key factor that better explains the higher OS rate in patients with a higher education level. This may indicate the presence of another unknown variable related to this as a protective or risk factor, such as social, environmental and biological variables.

Previous articles have linked successful treatment recovery to social and emotional support. Wynd et al. also describe infrequent TSE practice among men with decreased satisfaction with their current job assignment, diminished contentment with life in general, heightened concerns that disrupt daily life, more serious family problems involving spouses, children, or parents, and limited availability of supportive individuals to turn to [37–39]. On the other side, factors related to the Warburg effect might be the link to educational level, such as nutrition [40]. Focusing on TSE campaigns and promoting education could help reduce the OS rate. However, additional investigation is required to explore the matter and attain a more thorough understanding of its intricacies.

As a contrasting result, other sociodemographic variables that have been described as potential explanations for the situation among Hispanics and low-income countries (such as DTRH, patient delay, first manifestation, and patient residency) were not significant in either analysis model [12, 41, 42]. Although, it is important to mention that these are self-reported data captured through the clinical record. In line with the findings of Chertack et al., the presence of multiple barriers to TCa care, social determinants appear to be normalized in a stage-specific manner when patients are treated at a high-volume academic medical center specializing in the disease. However, a better understanding of these variables could be achieved by analyzing them in comparison to other populations or by focusing on non-seminomas [32, 43].

In contrast, the advanced stage at diagnosis exhibited consistent statistical significance across all four models using fatal outcome, treatment response, and chemo-response as dependent variables. Previous research has consistently underscored that Hispanic patients are often diagnosed at later disease stages [9, 44]. It has been stated that Mexican–American patients with testicular seminoma had poorer outcomes due to delayed diagnosis linked with advanced stages, potentially influenced by cultural factors [45]. Similarly, it was suggested that the increased fatality risk could be influenced by the cancer stage. However, underlying factors that contribute to these disparities within this ethnic group remain unidentified, encompassing disparities in exposures, tumor biology, treatments, or therapeutic response, attributing a 16% association with mortality to the stage itself [29].

The concept of susceptibility to cancer development across different stages is not novel. Demant et al. emphasized the importance of incorporating this notion into the assessment of biomarkers in preneoplastic lesions or early-stage cancer, which might be influenced by a genetic inclination to progress to a more advanced stage [26–28]. Additionally, the impact of genetic variations in drug metabolism and efficacy could also contribute to survival differences among Hispanics with advanced-stage tumors, potentially elucidating the connection between treatment response and chemo-response in our analysis [29].

Furthermore, the significance of disparities in histology subtypes within TGCT has been extensively discussed in various studies concerning the Hispanic population. Derouen et al. found no racial differences in OS among AYAs with seminoma, regardless of ethnicity [31]. However, Hispanics diagnosed with non-seminomas exhibited lower OS, even within the above median SEL category. Our results, as depicted comparing seminomas and non-seminomas, align with this data.

Supporting the estimation for the survival contrast by histology, the Cox regression analyzed the variables impacting survival time and found that non-seminomatous histology alone poses a risk for reduced survival, along with advanced stage and low SEL. Previous studies compared similar variables, such as patient delay and SEL, exclusively for non-seminomas, revealing significant associations. Thus, proposing that the divergent outcomes and treatment strategies observed in Hispanic and non-Hispanic white patients with TGCT might not solely be attributed to socioeconomic challenges and healthcare access disparities. These observed survival differences may also reflect intrinsic differences in tumor biology, prognosis, treatment availability, or even environmental exposure linked to histologic subtypes [25, 31, 46]. The fact that histology does not seem to explain the individual treatment response and chemo-response in our study, even when excluding sociodemographic factors (SEL and DTRH) as drivers of case-fatality rate also suggests the need for another type of treatment classification that involves histopathologic subtypes, biological and molecular profile of tumor development Medvedev et al., have proposed a molecular subclassification for seminomatous tumors that demonstrated an intrinsic tendency to exhibit a predictable chemo-response profile based on the genomic subtype characteristics. This tendency has been described in other types of cancers [25, 47–49].

However, it’s essential for further studies to be conducted within the Mexican population taking these factors into account. Notably, non-seminoma histology appears to be more prevalent among Hispanics than seminomas [8, 50], despite seminoma being the predominant presentation globally. Our results show the same trend observed in Hispanic research. Based on these findings, it seems plausible that interplay environmental exposures and socioeconomic disparities interact synergistically with genetic susceptibility or chemo-resistance driven by genomic instability [23, 24]. This leads to a more aggressive clinical outcome in the Hispanic population compared to other ethnicities with similar SEL. This underscores the vital need to enhance diversity in research participation [27–29].

**Fig. 3:**
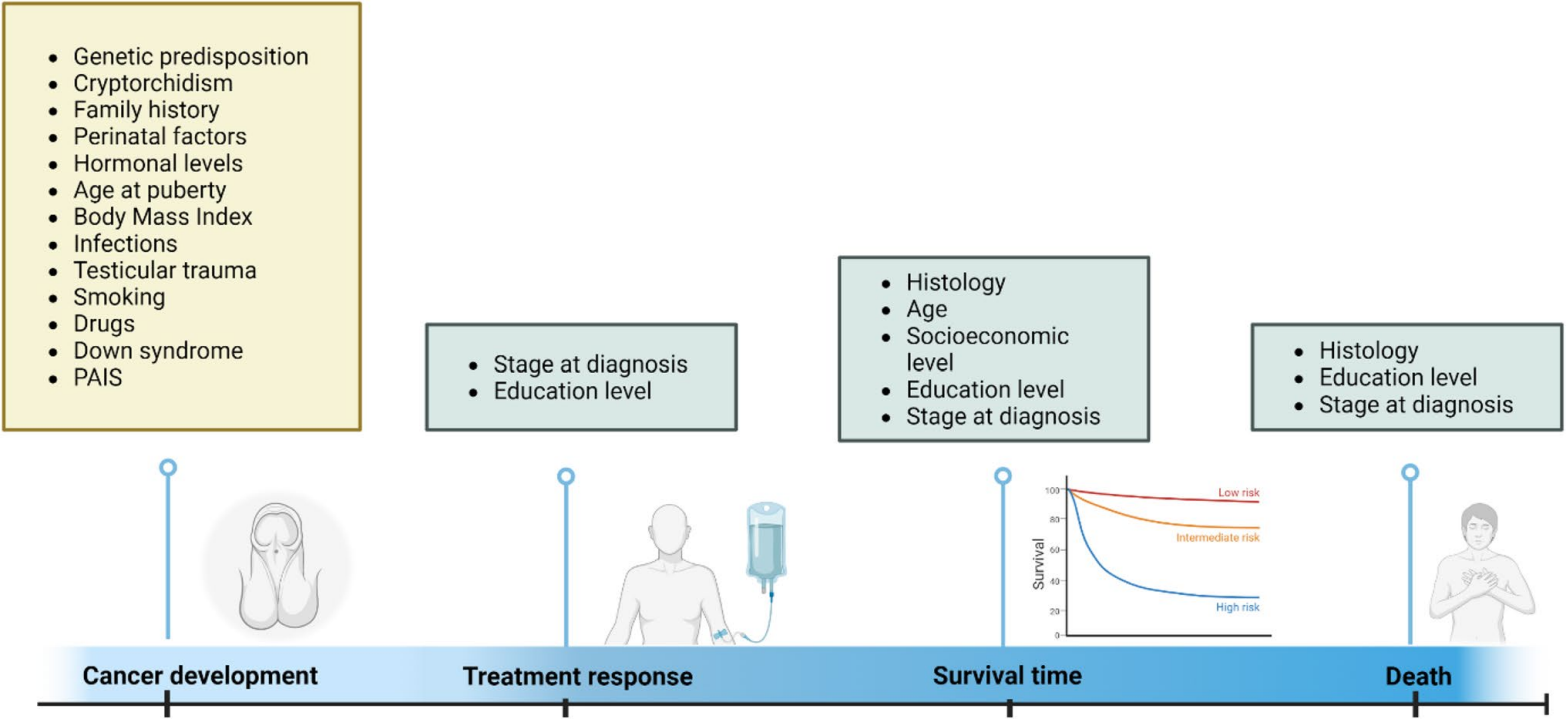
Potential TGCT risk factors among Mexican patients

Taken together, the lack of a significant impact of social determinants on disease lethality, summarized in Fig. 3, as discussed earlier, could provide signals about a potential factor unrelated to socioeconomic variables or related to these factors over time. Factors such as, genetic predisposition, physiological features (metabolism, inflammatory response, oxidative stress, drug response, etc.), clinical variables (cryptorchidism, family history, maternal and perinatal factors, hormonal levels, age at puberty, infections, testicular trauma, body mass index, etc.), lifestyle behaviors (nutrition, infections, drug exposure, sedentarism, etc.) and environmental exposures [27]. However, addressing these variables in the specific Hispanic population could provide a better understanding of the disease. In Mexico, suggestive data has been found linking TCa with genetic syndromes, but further research is needed [51, 52]. Promising investigation avenues in TCa could serve as a basis for understanding the exposome in Hispanics, specifically Mexicans. However, health disparities have hindered precision medicine research among understudied populations.

Research into TCa seems to be discouraged due to its favorable prognosis, along with various authors’ inferences about unfavorable outcomes due to socioeconomic factors among Hispanics [6, 12, 13, 22, 53, 54]. An inclusive approach that considers both epidemiological and genetic risk factors is essential for accurately evaluating individual risks of developing TCa and, consequently, customizing appropriate screening measures [27]. Improvements in diagnosis, treatment, and prognosis, are also possible. Novel approaches such as prevention, diagnostic and prognostic biomarkers, and radiomics may facilitate access to better treatment responses [28, 55]. However, at the moment, Mexico has limited research in these areas. Further research should be conducted, focusing on Hispanic populations worldwide, aiming to understand and identify genetic, epigenetic, and environmental factors that could explain the behavior of this cancer and develop precision medicine.

## Strengths and Limitations

This study presents valuable insights into TCa in Mexico and its association with sociodemographic variables. It is the first research to examine the epidemiological aspects of TCa among Mexican-Hispanic patients and compare the results with international perspectives on Hispanics. Due to the absence of a comprehensive national cancer database, the study had a small sample and was done in a single hospital center, making it challenging to identify cancer trends accurately to the entire Mexican or other Hispanic populations in different countries. However, despite the efforts to include patients from diverse regions, it is important to emphasize that our results are representative of the total hospital population in the period from 2007 to 2020 (data not shown). Moreover, the INCan treats patients from diverse socioeconomic backgrounds and regions, encompassing several central states of the Mexican Republic, from a radius of over 300 km, which could offer crucial information and significant indicators of health disparities around TCa. This level of regional coverage is uncommon for similar institutions worldwide, thus providing us with grounds to assert that our results are less prone to selection bias and may be reasonably representative of the central regions of Mexico. Larger and more representative samples are necessary to validate these findings.

While the study adjusted for multiple sociodemographic and clinical variables, there may still be unmeasured confounding factors influencing the observed associations. Lifestyle behaviors, environmental exposures, and healthcare access, beyond the variables included in the analysis, could contribute to the observed ethnic disparities [27]. Additionally, the retrospective study design limits establishing causal relationships between sociodemographic factors and TCa outcomes, warranting the interpretation of associations rather than causation.

To strengthen the evidence for causal relationships, prospective studies with longitudinal follow-up and comprehensive data collection are needed. Furthermore, incorporating qualitative research methods, such as interviews or focus groups, could provide valuable patient perspectives, and enhance our understanding of the sociodemographic factors influencing health disparities in Hispanic patients with TCa.

## Conclusion

In conclusion, the study revealed significant associations between non-seminoma histology, advanced stage at diagnosis, younger ages, and lower education levels with fatal cases of TCa. Additionally, lower education levels and advanced stages were linked to inadequate treatment responses. Moreover, Cox regression analysis indicated that non-seminoma histology, SEL, education level, advanced stage, and age significantly influenced survival time.

Our results suggest that the role of educational attainment could emerge as a key factor, justifying the onset of effective prevention strategies from basic educational levels for TCa among this population. In Hispanic-Mexican communities, TCa maintains a taboo status, underscoring the importance of TSE campaigns and promoting the development of emotional support networks in AYAs. The vigilance of patients with a low SEL could enhance our comprehension of associated risk factors that improve survival.

These findings emphasize the significance of considering sociodemographic determinants in understanding and addressing health disparities in TCa. However, by integrating sociodemographic, biological, and environmental factors, we can develop an analysis focusing on the tumor’s specific properties, from diverse perspectives (genomic, epigenomic, metabolomic, etc.) in understudied populations. This approach can be an effective strategy to reduce health inequalities through diverse precision medicine analysis methods, including specific biomolecules, immunotherapy, high-precision radiotherapy, drug repurposing, and advanced preventive measures like radiomics and radiogenomics. This, in turn, can lead to improved patient outcomes and promote health equity among Hispanic individuals diagnosed with TCa.

It is essential to address the study’s limitations and conduct further research to strengthen the evidence base and inform targeted interventions for this specific population. Long-term studies and comprehensive data collection are needed to strengthen evidence for causality. Qualitative research methods like interviews or focus groups can provide patient perspectives on sociodemographic factors influencing health disparities in Hispanic patients with TCa.

## Supplementary Information

Below is the link to the electronic supplementary material.

Supplementary file1 (DOCX 33 KB)

## Data Availability

The data that support the findings of this study are available on reasonable request from the corresponding author (R.G.-B: rodrigop@ciencias.unam.mx)
