# Supplementary material for: Understanding Sociodemographic Factors among Hispanics Through a Population-Based Study on Testicular Cancer in Mexico
Source: J Racial Ethn Health Disparities. 2023 Nov 14;12(1):148–60. doi: 10.1007/s40615-023-01859-0 (PMC11753316; doi:10.1007/s40615-023-01859-0)
Supplement: Supplementary file 1 — Supplementary file1 (DOCX 33 KB) [file 40615_2023_1859_MOESM1_ESM.docx]

**Supplementary Tables**

Supplementary Table 1. Criteria for the Assignment of Socioeconomic level

| CRITERIA FOR THE ASSIGNMENT OF SOCIOECONOMIC LEVEL | | | | | |
| --- | --- | --- | --- | --- | --- |
| The following six indicators are considered to the calculation of the 100% rating | | | | | |
| INDICATORS | PERCENTAGE | | | | |
| Family Income | 55% | | | | |
| Occupation | 10% | | | | |
| Feeding | 10% | | | | |
| Housing type | 18% | | | | |
| Place of residence | 5% | | | | |
| Family Health Status | 2% | | | | |
| Total | 100% | | | | |
| Family income is used as the basic indicator. The rest of the indicators affect the classification in a secondary way, but they allow us to have an idea of ​​the social and economic condition of the patient and his family. | | | | | |
| FAMILY INCOME. | | | | | |
| It refers to the sum of income received by the family, considering the total number of its members who are economically dependent on that income. | | | | | |
| FAMILY INCOME | NUMBER OF ECONOMIC DEPENDENTS | | | | |
|  | 1-2 | 3-4 | 5-6 | 7-8 | >9 |
|  | PUNCTUATION | | | | |
| Less than 1 GMW* | 0 | 0 | 0 | 0 | 0 |
| More than 1 GMW to 1.5 GMW | 15 | 10 | 5 | 0 | 0 |
| Over 1.5 GMW to 3.0 GMW | 20 | 15 | 10 | 5 | 0 |
| Over 3.0 GMW to 4.5 GMW | 25 | 20 | 15 | 10 | 5 |
| Over 4.5 GMW to 6.0 GMW | 30 | 25 | 20 | 15 | 10 |
| Over 6.0 GMW to 8.0 GMW | 35 | 30 | 25 | 20 | 15 |
| Over 8.0 GMW to 10.0 GMW | 40 | 35 | 30 | 25 | 20 |
| Over 10.0 GMW to 13.0 GMW | 45 | 40 | 35 | 30 | 25 |
| Over 13.0 GMW TO 16.0 GMW | 50 | 45 | 40 | 35 | 30 |
| 16.0 or More | 55 | 50 | 45 | 40 | 35 |
| *GMW: General Minimum Wage in force in Mexico City. | | | | | |
| OCCUPATION | | | | | |
| The type of occupation of the main economic provider of the family under study. | | | | | |
| POINTS | OCCUPATION | | | | |
| 0 | Unemployed | | | | |
| 1 | Unskilled workers | | | | |
| 2 | Retirees and pensioners | | | | |
| 3 | Scholar fellows | | | | |
| 4 | Officials, operators and craftsmen of mechanical arts and other trades | | | | |
| 5 | Service workers and vendors in shops and markets | | | | |
| 6 | Operators of machine installations and assemblers | | | | |
| 7 | Office employees, farmers, and skilled workers (agricultural and fishing) | | | | |
| 8 | Mid-level technicians and professionals | | | | |
| 9 | Scientific and intellectual professionals | | | | |
| 10 | Armed forces, executives, and investors | | | | |
| FEEDING | | | | | |
| PERCENTAGE OF FAMILY INCOME FOR FOOD | PUNCTUATION | | | | |
| 71% or More | 0 | | | | |
| 61% - 70% | 2 | | | | |
| 51% - 60% | 4 | | | | |
| 41% - 50% | 6 | | | | |
| 31% - 40% | 8 | | | | |
| 21% - 30% | 10 | | | | |
| The operationalization of this indicator must be consistent with the amount of income allocated to food. The social worker will determine the relationship's proportion and the individual will be deemed to have better economic capacity if they spend a smaller percentage of their income on meeting their nutritional demands. | | | | | |
| LIVING PLACE | | | | | |
| It refers to the place of living and its characteristics of a family or an individual. | | | | | |
| LIVING PLACE | PUNCTUATION | | | | |
| TYPE OF TENURE  Rented  Borrowed  Own paid * | 0  1  3 | | | | |
| INDOOR SERVICES  (Water, electricity, drainage, telephone)  0-1  2  3  4 or more | 0  1  2  3 | | | | |
| BUILDING EQUIPMENT  Cardboard, wood, or another sheet  Mixed  Masonry | 0  1  2 | | | | |
| NUMBER OF PEOPLE PER BEDROOM **  4 or more  3  1-2 | 0  1  3 | | | | |
| NUMBER OF ROOMS  1-2  3-4  5 or more | 0  2  3 | | | | |
| HOUSING TYPE ***  GROUP 1  (Social Protection Institution, round room, cave, hut or rural house, shack, slum)  GROUP 2  (Neighborhood, utility room)  GROUP 3  (Apartment or popular house) GROUP 4  (Apartment or middle-class house) GROUP 5  (Apartment or residential house) | 0    1    2    3    4 | | | | |
| TOTAL | 18 | | | | |
| *If it is in the payment process, it will be considered as rent.  **If there is more than one bedroom, the one with the largest number of people will be considered. | | | | | |
| PLACE OF RESIDENCE | | | | | |
| It refers to the geographical area in which the patient resides. | | | | | |
| GEOGRAPHIC AREA | PUNCTUATION | | | | |
| Mexico City  Mexico state  Other Federative Entities | 3  2  1 | | | | |
| LOCATION ZONE |  | | | | |
| Urban  Suburban Rural | 2  1  0 | | | | |
| TOTAL | 5 | | | | |
| FAMILY HEALTH STATUS | | | | | |
| It refers to the number of people who were chronically ill or receiving rehabilitation at the time the study was conducted and who cost money or reduced household income. | | | | | |
| NUMBER OF SICK PEOPLE IN THE FAMILY NUCLEUS | PUNCTUATION | | | | |
| Three or the main contributor to the family income. | 0 | | | | |
| Two | 1 | | | | |
| One (includes patient) | 2 | | | | |
| ASSIGNMENT OF SOCIOECONOMIC CLASSIFICATION | | | | | |
| The final score will be calculated when the values of all the indicators have been obtained, indicating which socioeconomic category the user corresponds to. | | | | | |
| PUNCTUATION | SOCIOECONOMIC CLASSIFICATION  (0-6) | SOCIOECONOMIC CLASSIFICATION  (Below median, above median) | | | |
| 0-15 | 0 | Below median | | | |
| 16-24 | 1 |  |  |  |  |
| 25 - 36 | 2 |  |  |  |  |
| 37 - 52 | 3 | Above median | | | |
| 53 - 68 | 4 |  |  |  |  |
| 69 - 84 | 5 |  |  |  |  |
| 85 - 100 | 6 |  |  |  |  |

Supplementary Table 2. Complete univariate analysis of Fatal and Non-Fatal Cases

|  | **Fatal cases** | **Non-fatal cases** | OR (95%CI) | P-Value^a^ |
| --- | --- | --- | --- | --- |
|  | n= 49 (20.1%) | n= 195 (79.9%) |  |  |
| **Age in years**, median (IQR) | 25 (21-29) | 26 (22-32) | - | 0.24^b^ |
| **Delay in months,** median (IQR) | 3 (2-7) | 3 (1.4-7) | - | 0.78^b^ |
| **DTRH,** median (IQR) | 44 (21-82) | 34 (15.8-70) | - | 0.15^b^ |
| **Patient residency**  Local  Out-of-town | 17 (34.69)  32 (65.31) | 87 (44.62)  108 (55.38) | Ref  1.52 (0.76-3.11) | 0.21 |
| **Histology**  Seminoma  Non-seminoma | 3 (6.12)  46 (93.88) | 90 (46.15)  105 (53.85) | Ref  13.14 (3.98-67.75) | <0.001^c^ |
| **Stage**  Locoregional  Advanced | 3 (6.12)  46 (93.88) | 150 (76.92)  45 (23.08) | Ref  51.11 (15.04-263.91) | <0.001^c^ |
| **Initial symptom**  Testicular pain  Testicle swelling  Mixed symptoms  Metastasis related | 1 (2.04)  19 (38.78)  8 (16.33)  21 (42.86) | 15 (7.69)  113 (57.95)  49 (25.13)  18 (9.23) | Ref  2.52 (0.29-19.27)  2.44 (0.28-21.73)  17.5 (1.49-166.90) | 0.70^c^  0.67^c^  <0.001^c^ |
| **Treatment modality**  Local (orchiectomy)  Systemic (chemotherapy) | 4 (8.16)  45 (91.84) | 52 (26.67)  143 (73.33) | Ref  4.10 (1.38-16.35) | <0.05^c^ |
| **Treatment response**  Complete  Inadequate | 1 (2.04)  48 (97.96) | 174 (89.23)  21 (10.77) | Ref  397.71 (59.12-16112.10) | <0.001^c^ |
| **Chemo-response**  Sensitive  Resistant | 1/45 (2.22)  44/45 (97.78) | 127/143 (88.81)  16/145 (11.19) | Ref  349.25 (49.76-14202.19) | <0.001^c^ |
| **Socio-economic level**  Above median  Below Median | 9 (18.37)  40 (81.63) | 71 (36.41)  124 (63.59) | Ref  2.54 (1.13-6.30) | <0.05 |
| **Perceived socio-economic level**  Above median  Below Median | 9 (18.37)  38 (81.63) | 69 (36.41)  128 (63.59) | Ref  2.27 (1.040-4.982) | <0.05 |
| **Education level**  Primary  Middle school  High School  Higher | 18 (36.73)  8 (16.33)  19 (38.78)  4 (8.16) | 18 (9.23)  62 (31.79)  58 (29.74)  57 (29.23) | Ref  0.13 (0.04-0.38)  0.33 (0.14-0.78)  0.07 (0.02-0.28) | <0.001  <0.01  <0.001^c^ |

Supplementary Table 3. Logistic Regression Analysis of Treatment and Chemo-response in TGCT patients.

|  | **Treatment response** ^a^ | | **Chemo-response** ^a^ | |
| --- | --- | --- | --- | --- |
|  | Adjusted OR (95% CI) | p-value | Adjusted OR (95% CI) | p-value |
| **Histology**  Seminoma  Non-seminoma | Ref  0.962 (0.335 - 2.762) | -  0.942 | Ref  1.738 (0.562 - 5.375) | -  0.338 |
| **Socioeconomical Level**  Above median  Below median | Ref  1.526 (0.585 - 3.982) | -  0.388 | Ref  1.251 (0.449 - 3.490) | -  0.669 |
| **Education level**  Elementary  Middle school  High School  Higher | Ref  0.343 (0.103 - 1.138)  0.609 (0.177 - 2.097)  0.120 (0.027 - 0.529) | -  0.080  0.432  <0.05* | Ref  0.551 (0.168 - 1.802)  1.607 (0.457 - 5.651)  0.256 (0.059 - 1.118) | -  0.324  0.459  0.070 |
| **Stage**  Locoregional (Stage I, II)  Advanced (Stage III) | Ref  29.02 (11.421 - 73.739) | -  <0.001** | Ref  20.29 (6.651 - 61.944) | -  <0.001** |
| **DTRH** | 1.002 (0.998 - 1.006) | 0.308 | 1.003 (0.999 - 1.007) | 0.124 |
| **Patient delay** | 0.986 (0.955 - 1.019) | 0.410 | 0.976 (0.941 - 1.012) | 0.193 |
| **Age** | 0.974 (0.915 - 1.036) | 0.399 | 1.001 (0.941 - 1.065) | 0.982 |

* Significant at p <0.05 ** Significant at p <0.001

**Supplementary Table 4.** Diagnostic Delay by Educational Levels

| **Education Level** | **Median Patients Delay to Diagnostic (months)** |
| --- | --- |
| Basic  Middle School  High School  Higher Education | 6.05  5.53  5.49  8.01 |
| Kruskal-Wallis | p-value= 0.196 |
